# TRPM8 overexpression suppresses hepatocellular carcinoma progression and improves survival by modulating the RTP3/STAT3 pathway

**DOI:** 10.1002/cam4.70109

**Published:** 2024-10-09

**Authors:** Lichan Chen, Nansong Xu, DongMei Gou, Jianning Song, Mingqin Zhou, Yajun Zhang, Haohua Zhang, Liwen Zhu, Weihong Huang, Yue Zhu, Cheng Gao, Dayong Gu, Yong Xu, Hongzhong Zhou

**Affiliations:** ^1^ Department of Laboratory Medicine The First Affiliated Hospital of Shenzhen University, Shenzhen Second People's Hospital; Shenzhen Key Laboratory of Medical Laboratory and Molecular Diagnostics; Guangzhou Medical University Shenzhen China; ^2^ State key laboratory of Oncology in South China, Collaborative Innovation Center for Cancer Medicine Sun Yat‐sen University Cancer Center Guangzhou China; ^3^ Shenzhen Third People's Hospital Southern University of Science and Technology Shenzhen China; ^4^ Department of Critical Care Medicine Cancer Hospital of Shantou University Medical College Shantou China; ^5^ Guangzhou University of Chinese Medicine Guangzhou China; ^6^ Department of Pathology, Guangdong Provincial People's Hospital (Guangdong Academy of Medical Sciences) Southern Medical University Guangzhou China; ^7^ Medicine Department of Biochemistry and Molecular Biology Medical College of Jinan University Guangzhou China

**Keywords:** hepatocellular carcinoma, TRPM8, RTP3/STAT3 pathway, AD80

## Abstract

**Background and Aims:**

Hepatocellular carcinoma (HCC) is a malignant tumour associated with high morbidity and mortality rates worldwide. Recently, TRPM8 was reported to play an important role in tumour progression. However, the precise role of TRPM8 in HCC remains unclear. In this study, we explored the expression levels, molecular functions and underlying mechanisms of TRPM8 in HCC.

**Methods:**

Tissue samples were used to analyse the expression of TRPM8 to assess its diagnostic value for prognosis. Cell Counting Kit‐8, EdU and colony formation assays were performed to evaluate the effects of TRPM8 on cell proliferation, whereas the Transwell assay was used to assess cell migration and invasion. The role of TRPM8 in vivo was evaluated using a mouse subcutaneous xenograft tumour model. We performed PPI network analyses to understand the possible mechanisms of TRPM8 action.

**Results:**

TRPM8 expression was decreased in HCC tissues and was correlated with histological grade and poor patient prognosis. Functionally, TRPM8 repressed the proliferation and metastasis of HCC cells both in vitro and in vivo by modulating the RTP3/STAT3 signalling pathway.

**Conclusion:**

Our findings underscore the critical role of the TRPM8‐RTP3‐STAT3 axis in maintaining the malignant progression of HCC. Moreover, our study demonstrates that AD80 is involved in anti‐tumour processes by upregulating the expression of TRPM8.

## Graphical abstract

TRPM8 is associated with various tumours, and in HCC, its low expression may play a critical role in disease progression through the TRPM8‐RTP3‐STAT3 axis. The multikinase inhibitor AD80 significantly upregulates TRPM8 expression, which in turn inhibits the proliferation and invasion of HCC cells by modulating the RTP3/STAT3 pathway. This suggests that the TRPM8‐RTP3‐STAT3 axis may be a key factor in HCC pathogenesis, and AD80 could contribute to the anti‐tumour process by enhancing TRPM8 expression.

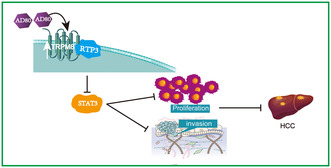

## 1 INTRODUCTION

According to global cancer statistics in 2018, liver cancer ranked as the sixth most common cancer worldwide, with the fourth highest mortality rate. Approximately 841,000 new cases and 782,000 deaths occur each year due to this malignancy. Primary liver cancer includes hepatocellular carcinoma (HCC) (75%–85%), intrahepatic cholangiocarcinoma (10%–15%) and other rare subtypes.^1^ Although monitoring patients with liver cirrhosis can be used to diagnose early liver tumours, most liver cancers are diagnosed at an advanced stage, leading to a poor prognosis for many patients.^2^ Several treatment methods are being tested in clinical trial.^3^ Due to the characteristics of high recurrence and easy metastasis of liver cancer, the treatment of liver cancer is still full of challenges. Although AFP can be a diagnostic marker for liver cancer, its sensitivity and specificity are not high.^4^ Moreover, AFP cannot accurately diagnose early‐stage liver cancer. Therefore, there is an urgent need to identify new diagnostic markers for liver cancer to enhance the diagnosis rate and improve the prognosis for patients with liver cancer.

The TRPM family comprises eight different channels (TRPM1‐TRPM8), which play important roles in processes related to temperature,^5^ taste,^6^ oxidative stress induction,^7^ myogenic response,^8^ ion homeostasis,^9^ vascular tone regulation^10^ and cell death.^11^ Transient receptor potential cation channel subfamily M member 8 (TRPM8) is a non‐selective cation channel that controls calcium homeostasis. It is considered the main thermoreceptor for cells and behaviours in response to cold stimuli and has advanced to the clinical stage of drug development.^12^ It was first found that the expression of TRPM8 was mainly limited to the prostate and was upregulated in prostate cancer.^13^ Current studies have shown that TRPM8 is not only expressed in prostate cancer but is also widely abnormally expressed in a variety of tumours, including pancreatic cancer, melanoma, breast cancer, bladder cancer, osteosarcoma and squamous cell carcinoma. This abnormal expression affects tumour proliferation, apoptosis, migration, invasion, metastasis and other biological behaviours.^14^ Because TRPM8 plays an important role in tumourigenesis and cancer progression, it has potential value in diagnosing and treating cancer and may become a new molecular target. However, the role and mechanisms of TRPM8 in liver cancer remain unexplored. Thus, whether TRPM8 can be used as a new molecular target in liver cancer is worth exploring.

In the present study, we investigated the expression, clinical significance and potential biological functions of TRPM8 in HCC. Furthermore, cell function experiments showed that the overexpression of TRPM8 contributed to suppressing the proliferation and metastasis of HCC cells in vitro and in vivo by inhibiting the receptor transporter protein 3 (RTP3), is a member of the transmembrane proteins family (TMEMs).^15^, ^16^ More importantly, AD80, a multikinase inhibitor, significantly promoted the expression of TRPM8 and inhibited HCC cells proliferation and metastasis, suggesting that AD80 may be promising for the treatment of HCC patients.

## 2 MATERIALS AND METHODS

### 2.1 Cell culture

Available HCC cell lines (HUH‐7, PLC/PRF/5, SNU‐449 and HLE) were obtained from the American Type Culture Collection (ATCC). Cells were cultured according to the manufacturer's instructions. All four HCC cell lines were cultured in Dulbecco's Modified Eagle Medium (DMEM) (Cytiva/Global Life Sciences Solutions, Marlborough, MA) containing 10% foetal bovine serum (FBS) (Gibco, New York, USA), and kept at 37°C, 5% CO_2_.

### 2.2 Plasmid extraction

The SteadyPure plasmid DNA Extraction Kit (AG21001, ACCURATE BIOTECHNOLOGY, HUNAN) was used to extract TRPM8 plasmid DNA.

### 2.3 Western Blot

First, protein concentration was determined using the BCA protein detection method. Subsequently, a protein glue of 1.5 mm was prepared, and the western blot 10 × electrophoresis solution was diluted to 1 × with ddH20. The transmembrane solution was prepared by mixing 10 × transmembrane solution, methanol and ddH2O at a ratio of 1:2:7 and stored at 4°C. Following SDS‐PAGE, the protein was added to the gel (120 V for 80 min). After electrophoresis, the proteins were transferred to a configured transmembrane solution and a PVDF membrane (200 mA 90 min). At the end of the film transfer, the PVDF film in the corresponding area was cut and placed in a cartridge containing 5% skimmed milk to seal the membrane for 1 h. After cleaning the membrane with TBST solution, the corresponding antibody was added and incubated overnight at 4°C. The next day, after incubating the membrane with the secondary antibody for 1.5 h, the membrane was reacted with a suitable concentration of chromogenic solution, and a development image was obtained. HRP substrate luminol reagent (Millipore, Massachusetts, USA) were utilised for imaging. Primary antibodies against TRPM8 (Zen‐bioscience, Chengdu, China), RTP3 (Epigentek, USA), STAT3 (Proteintech, Wuhan, China) and GAPDH (Proteintech, Wuhan, China) were employed.

### 2.4 Immunohistochemistry Analysis

First, paraffin blocks were sliced and placed in an oven for fixation. Fixed paraffin sections were de‐paraffinised by immersion in xylene. Afterwards, the paraffin sections were soaked successively in 100% ethanol, 90% ethanol, 80% ethanol and 70% ethanol for hydration. After washing with tap water and PBS (Cytiva), the paraffin sections were put in 1 × EDTA for 20 min, cooled at room temperature for 1 h for antigen repair, and washed with PBS. Endogenous peroxidase blockers were used for blocking (37°C, 10 min), and after washing, the diluted primary antibody against TRPM8 (Zen‐bioscience, Chengdu, China) was added and incubated overnight at 4°C. After PBS washing, the reaction enhancer (37°C, 30 min) was added to the paraffin section the following day. Then, a sufficient amount of enhanced enzyme labelled goat anti‐mouse/rabbit IgG polymer (37°C, 30 min) was added. After DAB chromogenic agent staining, haematoxylin staining and hydrochloric acid alcohol differentiation, the paraffin sections were soaked in 80% ethanol, 90% ethanol, 100% ethanol and xylene. Finally, the film was sealed with neutral gum, observed under a microscope and photographed (Carl Zeiss AG, Germany). The assessment of the intensity of immunohistochemical staining was based on Image J (java, National Institutes of Health). The intensity of staining can be scored as 1 (no staining), 2 (weak staining), 3 (moderate staining) and 4 (strong staining). The total number of cells in each field and the number of positive staining cells at each intensity level were counted. Immunohistochemistry (IHC) Analysis kit (Zsgb‐Bio, Beijing, China) was employed.

### 2.5 Quantitative Real‐time PCR

Total RNAs were extracted using TRIzol reagent (AG, Hunan, China). The cDNAs were synthesised from 1 μg RNA using the M‐MLV 4 First‐Strand cDNA Synthesis Kit (Biomed, Beijing, China). According to the product instructions, quantitative real‐time PCR (qRT‐PCR) was performed using Green qRT‐PCR MasterMix (Biomed, Beijing, China) according to the manufacturer's instructions. Amplification was performed using a QuantStudio 3 Real‐Time PCR System (Thermo Fisher Scientific, Massachusetts, USA). GAPDH was used as an internal reference to normalise gene expression. Data analysis was based on the 2^−ΔΔCt^ formula. The primers used in this study have been listed (Table S1).

### 2.6 Cell transfection

Specific shRNAs targeting TRPM8 were transfected with TRPM8‐RNAi lentiviruses (Genechem Co., LTD, Shanghai, China) to generate TRPM8 silencing HUH7 and PLC/PRF/5 cells. 1.5 × 10^4^ cells were inoculated into 24‐well plates, and infection conditions with an MOI of 50 were selected. Corresponding reagents were added according to the instructions, and cells were transfected for 16–20 h. The transfection supernatant was discarded, and green fluorescence was observed 72 h later under an inverted fluorescence microscope (Carl Zeiss AG, Germany). TRPM8 cDNAs were subcloned into the pcDNA3.1‐EGFP‐C2 plasmid to generate TRPM8 pcDNA3.1‐EGFP‐C2.

### 2.7 Colony formation assays

The four HCC cell lines were seeded in 6‐well plates at a density of 1 × 10^3^ cells per well and incubated for 14 days. They were then fixed with 4% paraformaldehyde and stained with 0.01% crystal violet (Servicebio, Wuhan, China) for 2 h. The colonies were counted under a microscope (Carl Zeiss AG).

### 2.8 Cell Counting Kit‐8 (CCK‐8) cell proliferation assays

Cell viability was determined using the CCK‐8 assay (MedChem Express, MCE USA). Briefly, 2 × 10^4^ and 8 × 10^3^ plasmid‐transfected SNU‐449 and HLE cells were inoculated into 96‐well plates of DMEM medium containing 10% FBS for 0, 24, 48, 72 and 96 h, respectively. Then 10 μL CCK‐8 solution was added and incubated at 37°C, 5% CO_2_ for 2 h. Absorbance was measured at 450 nm to assess cell viability.

### 2.9 EdU (5‐ethynyl‐2‐deoxyuridine) cell proliferation assays

SNU‐449 and HLE cells infected with plasmids or lentiviruses were inoculated into 24 well plates at a density of 5 × 10^4^ cells per well for the EdU assay. Equal volumes of EdU working solution were added to each well, and the cells were cultured at 37°C, 5% CO_2_ for 2 h. The cells were then fixed with 4% neutral paraformaldehyde for 30 min, incubated with 0.1% Triton X‐100‐PBS (Sigma‐ Aldrich, St. Louis, Missouri, USA) for a minimum of 10 min, and washed once with PBS for 5 min at room temperature. Next, 100 μL of 1 × Apollo staining reaction solution was added to each well, incubated at room temperature, and away from light for 30 min on a shaker. Nuclei were stained with Hoechst 33342. The tablets were sealed with an antifluorescence quencher and observed under an inverted fluorescence microscope (Carl Zeiss AG). EdU in vitro assay kit (Ribo‐Bio, Guangzhou, China) was employed.

### 2.10 Transwell migration assays

Transwell migration experiments were conducted in TRPM8 knockout and overexpressed HCC cells to estimate the migratory capacity of SNU‐449, HLE, HUH‐7 and PLC/PRF/5 cells. Transfected cell suspensions (1 × 10^5^ cells) were resuspended in 350 μL of serum‐free media and inoculated in the upper chambers. Cell migration was induced by introducing a 10% FBS cell medium into the lower chambers (Corning/Costar, New York, USA). After incubation for 15 h, the cells were fixed with 4% paraformaldehyde (Servicebio) at room temperature for 25 min, washed twice with PBS, and stained with 0.1% crystal violet solution (Biyotime, Shanghai, China) at room temperature for 15 min. After drying at room temperature, the migrating cells were observed using an inverted optical microscope (Carl Zeiss AG). Five random fields were photographed, and the number of cells in these fields was calculated and averaged.

### 2.11 Matrigel invasion assays

To evaluate the invasive ability of HLE and HUH‐7 cells, the upper chambers of the Transwell chamber (Corning/Costar) were coated with Matrigel (Corning/Costar). The transfected HCC cells (1.5 × 10^5^ cells) were resuspended in 300 μL of serum‐free high glucose DMEM medium (Cytiva) and inoculated in the upper chambers. Cell invasion was induced by adding 900 μL of medium containing 15% FBS in the lower chambers. After induction at 37°C for 15 h, the cells were fixed with 4% paraformaldehyde at room temperature for 25 min, washed twice with PBS, and stained with 0.1% crystal violet solution at room temperature for 15 min. After drying at room temperature, the invasive cells were observed using an inverted optical microscope (Carl Zeiss AG). Five random fields were photographed, and the number of cells in these fields was calculated and averaged.

### 2.12 Calcium imaging

Intracellular Ca^2+^ concentrations were determined using the ratiometric dye Furo‐8/AM (AAT Bioquest, USA). Cells were loaded with a mixture of 4 μM Fluo‐8/AM in 20% Fluronic F‐127 (SIGMA, USA) at 37°C, 5%CO_2_ for 20 min. They were then washed and immersed in the extracellular solution, which contained 126 mM NaCl, 5.4 mM KCl, 1 mM CaCl_2_, 1.2 mM MgCl_2_, 10 mM N‐(2‐hydroxyethyl)‐piperazine‐N′‐ethanesulfonic acid (HEPES), 10 mM glucose (NaOH to pH 7.4). Observation were performed on a Zeiss LSM 800 laser scanning confocal microscope using a 63× objective. Images and data were collected using the companion ZEN software. GraphPad Prism 9.3.0 software was used to analyse the data.

### 2.13 Tumour xenograft mouse model

Male BALB/c nude mice, aged 4–5 weeks, were purchased from Weitong Lihua Experimental Animal Technology Co., Ltd. (Beijing, China). Briefly, animal subcutaneous xenograft tumour models were established by the subcutaneous injection of 5 × 10^6^ SNU‐449 cells stably overexpressing TRPM8 into the thighs of the nude mice. Tumour growth was measured every 7 days using a Vernier calliper (Deli, Zhejiang, China). The tumour size was calculated using the following formula: volume = (length×width^2^)/2. On day 28, mice were sacrificed, and tumours were excised, weighed, photographed and fixed in 4% paraformaldehyde for further experiments. All animal experiments were approved by the Shenzhen Second People's Hospital (Shenzhen, China), and all procedures were performed in accordance with the institutional guidelines.

### 2.14 Integrative Molecular Database of Hepatocellular Carcinoma

The Integrative Molecular Database of Hepatocellular Carcinoma (HCCDB, http://lifeome.net/database/hccdb) is a one‐stop online resource for exploring HCC gene expression. The database contains 15 datasets and 3917 samples, integrates data from GTEx and TCGA, and defines a 4D index based on log_2_ fold change (FC) to comprehensively summarise gene expression pattern.^17^ We used data from the HCCDB to evaluate the differences in the expression of the selected gene (TRPM8) between HCC and adjacent tissues.

### 2.15 MERAV

The metabolic gene rapid visualiser (MERAV, http://merav.wi.mit.edu) is based on human gene table data obtained from the NCBI GEO repository, which reflects the expression of human genes in normal tissues, cancer cell lines and primary tumours.^18^ We used this database to analyse differences in the expression of TRPM8.

### 2.16 The Human Protein Atlas

The Human Protein Atlas (HPA; https://www.proteinatlas.org/) analyses the human genome at different levels, including organs, tissues, cells and organelle.^19^ We used HPA to analyse the expression of TRPM8 in HCC, normal tissues and cells.

### 2.17 UALCAN

UALCAN (http://ualcan.path.uab.edu/)is an online database based on relevant tumour data from the TCGA database. The database can analyse relative gene expression in tumour and normal samples and clinicopathological parameters such as individual cancer stage, tumour grade, race and body weight. Additionally, it can evaluate the effects of gene expression levels and clinicopathological characteristics on patient survival.^20^ We used this database to analyse the differences in TRPM8 expression between normal subjects and patients with HCC with different pathological parameters.

### 2.18 Gene Expression Profiling Interactive Analysis

Gene Expression Profiling Interactive Analysis (GEPIA, http://gepia.cancer‐pku.cn/) is an online database based on TCGA and GTEx data. GEPIA provides key interactive and customisable functions, including seven main tabs: general, differential genes, DIY expression, survival, similar genes, correlation and PCA. These functions enable differential expression analysis, mapping, correlation analysis, patient survival analysis, similar gene detection and dimensionality reduction analysis.^21^ TRPM8 was analysed using the GEPIA database to explore differences in its expression in patients with HCC.

### 2.19 Kaplan–Meier Plotter

The Kaplan–Meier Plotter (http://kmplot.com/analysis/) is an online database based on gene expression data and survival information of cancer patients in the TCGA database.^22^ It can be used to evaluate differential expression in progression‐free survival (PPS), recurrence‐free survival (RFS) and overall survival (OS) among cancer patient.^23^ Information on liver, breast, ovarian, lung and gastric cancers can also be obtained. The median mRNA expression level, HR, 95% CI and *p*‐values related to liver cancer can be found in this database. When the *p*‐value was <0.05, a statistically significant difference was considered.^24^ We used the Kaplan–Meier Plotter to analyse the prognosis of TRPM8 in liver cancer samples and the effect of differences in high and low TRPM8 expression on OS in patients with liver cancer.

### 2.20 cBioPortal

The cBio Cancer Genomics Portal(http://www.cbioportal.org) is an open‐access resource for interactively exploring multidimensional cancer genome datasets, providing data from over 5000 tumour samples from 20 cancer studies.^25^ The database can be queried across all TCGA projects and published literature. Its functions include graphical summarisation of gene‐level data across multiple platforms, correlation analysis between genes or other data types, query and analysis of single genes, co‐expression analysis of selected genes, enrichment analysis, survival analysis, mutual exclusion analysis and visualisation of each patient's data.^26^ We selected TRPM8 in patients with liver cancer for data analysis.

### 2.21 LinkedOmic

The LinkedOmics database (http://www.linkedomics.org) contains multiple sets of clinical data from 32 cancer types and 11,158 patients from the TCGA database. This is the first multigroup database to integrate mass spectrometry (MS) global proteomic data from selected TCGA tumour samples generated by the Clinical Proteomics Oncology Analysis Alliance (CPTAC).

LinkedOmics has three data‐analysis modules: LinkFinder, LinkCompare and LinkInterpreter. The LinkFinder module was used to study the association between molecular or clinical attributes and all other attributes in the selected cancer cohort. The LinkCompare module can be used to compare associations identified by LinkFinder with different attributes within the same target dataset or with the same attributes, tumour types or tumour subtypes across target datasets from different taxonomic platforms. The LinkInterpreter module converts the association between the LinkFinder and LinkCompare recognition into biological understanding. This module uses overexpression and gene set enrichment analyses for gene set and pathway analysis.^27^ We used RNA‐seq data from a liver cancer database and the Pearson correlation test to analyse the biological function of TRPM8.

### 2.22 STRING

The Search Tool for the Retrieval of Interacting Genes (STRING, https://cn.string‐db.org) aims to collect, store and integrate all publicly available sources of information on protein–protein interactions and supplement this information by calculating predictions. The STRING database contains extensive and diverse benchmark data sources, spanning over 5090 organisms and reaching 24.6 million proteins.^28^ We used this database to analyse the interactions between TRPM8 proteins in HCC.

### 2.23 Statistical analysis

All experiments were independently replicated at least three times. Statistical analyses were conducted using GraphPad Prism 9.3.0 (GraphPad Software, Inc., La Jolla, CA, USA). Comparisons were made using the Student's *t*‐test or ANOVA and Bonferroni's post‐hoc test. *p < 0.05 was considered as the criterion for statistical significance.

## 3 RESULTS

### 3.1 Expression Analysis of TRPM8 in Various Databases

**FIGURE 1:**
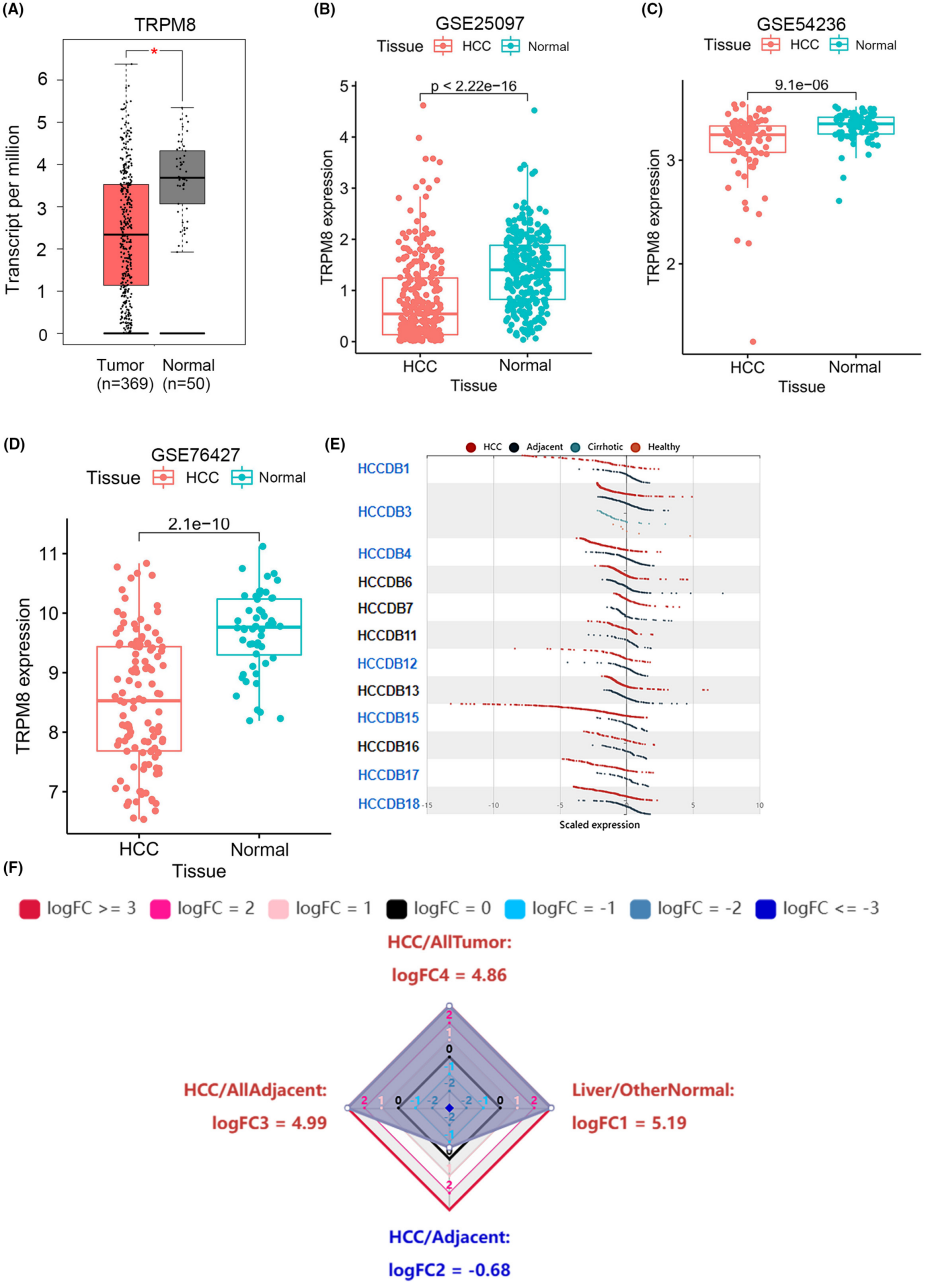
TRPM8 expression in different datasets. (A) The mRNA expression level of TRPM8 was lower in HCC cells and Primary liver tissues than that in normal tissues in the MERAV. (B) The expression of TRPM8 in GEO database dataset GSE25097. (C) The expression of TRPM8 in GEO database dataset GSE54236. (D) The expression of TRPM8 in GEO database dataset GSE76427.(E) The expression of TRPM8 in different HCC datasets. (F) Radar map of TRPM8 overall expression among different types of tissues.

First, we determined the expression of TRPM8 in different organisations by analysing the MERAV database. TRPM8 expression was much higher in normal tissues (Mean ± SEM, 3.56 ± 0.52) (*n* = 50) than in HCC (Mean ± SEM, 2.59 ± 0.97) (*p* < 0.05; *n* = 369) (Figure 1A). Consistent with the above data, we found that TRPM8 expression in the GSE25097, GSE54236 and GSE76427 datasets was higher in normal tissues than in liver cancer tissues (Figure 1B–D). In GSE25097 dataset, the Mean ± SEM of normal tissues (*n* = 243) and tumour tissues (*n* = 268) were 1.45 ± 0.04 and 0.80 ± 0.05, respectively (*p* < 0.0001). Similarly, Mean ± SEM of normal tissues (*n* = 80) and tumour tissues (*n* = 81) were 3.32 ± 0.02 and 3.15 ± 0.03 in GSE54236 dataset (*p* < 0.0001). And in GSE76427 dataset, the Mean ± SEM of normal tissues (*n* = 52) and tumour tissues (*n* = 115) were 9.68 ± 0.09 and 8.53 ± 0.10, respectively (*p* < 0.0001). Consistent with the above data, we also compared different liver cancer datasets in the HCCDB database and found 12 datasets that supported the above results (Figure 1E). As shown in Figure 1F, we determined the expression of TRPM8 in the HCC dataset using the HCCDB database and found that the level of TRPM8 in HCC tissues was much lower than that in adjacent tissues (HCC/adjacent tissues: logFC = −0.68).

In addition, HPA database analysis revealed that the expression of TRPM8 in normal liver tissues was higher than that in most human tissues (Figure S1A). In contrast, in cancer samples, the expression of TRPM8 in HCC tissues was very low (Figure S1B). Genetic changes in TRPM8 were studied using the CBioPortal database, and mutations in TRPM8 were analysed using log RNA Seq V2 RSEM data from the liver cancer database. Among 360 patients with HCC, nine (2.5%) had TRPM8 gene alterations, and 105 (29.17%) had TP53 gene mutations. The results showed one amplification mutation, one truncating mutation, one splice mutation and six missense mutations (Figure S2A,B). These results suggested that TRPM8 expression is consistently downregulated in human HCCs.

### 3.2 Low expression of TRPM8 is associated with clinicopathological features and poor survival of HCC patients

**FIGURE 2:**
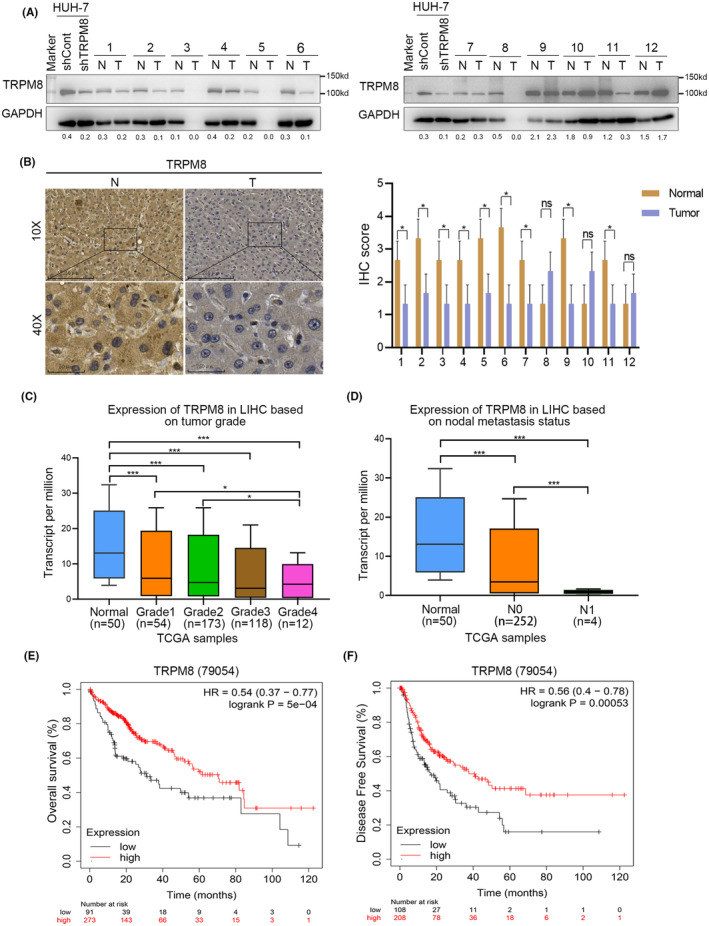
Relationship between TRPM8 expression and clinical value in hepatocellular carcinoma. (A) Western blot results showed that TRPM8 protein level in HCC tissues was lower than that in paired normal tissues. (B) TRPM8 expression in HCC adjacent tissues and tumour tissues tested by IHC. (C)The expression of TRPM8 was negatively correlated with tumour grade. (D)The expression of TRPM8 was negatively correlated with nodal metastasis status. (E) The low expression of TRPM8 in Kaplan–Meier Plotter is associated with poor overall survival. (F) The low expression of TRPM8 in Kaplan–Meier Plotter is associated with disease free survival. Scale bars: 250 μm. *p < 0.05.

To further verify the clinical relevance of TRPM8 in HCC, the expression of TRPM8 in 12 pairs of tissue samples was evaluated using western blotting. The results showed that the expression of TRPM8 in nine pairs of tissues (75%) was lower than that in normal tissues (Figure 2A). Furthermore, the IHC assay confirmed that the expression of TRPM8 protein in the cytoplasm was higher in normal tissues than in tumour tissues (Figure 2B), which is consistent with our previous findings. The UALCAN database was used to evaluate the influence of gene expression levels and clinicopathological characteristics on the patients. In HCC, TRPM8 expression was negative for tumour grade (Figure 2C) and nodal metastasis status (Figure 2D). These data suggest that TRPM8 is underexpressed in HCC, leading to poor clinical outcomes. To analyse the relationship between TRPM8 and the survival rate of patients with liver cancer, we used the Kaplan–Meier Plotter based on the TCGA database. The results showed that among HCC patients, patients with low TRPM8 expression had poor OS (Figure 2E, *p* = 5e‐04). Meanwhile, low TRPM8 expression was also positively correlated with progression‐free survival (Figure 2F, *p* = 0.00053). These results suggest that TRPM8 is closely related to the poor prognosis of HCC patients.

Furthermore, to evaluate the influence of TRPM8 gene expression levels and clinicopathological characteristics, the UALCAN database was used. TRPM8 expression in HCC tissues negatively correlated with tumour grade (Figure S3A). Database analysis showed that TRPM8 negatively correlated with patient race (Figure S3B) and weight (Figure S3C). Differences among patients of different sexes and ages were compared (Figure S3D,E).

### 3.3 TRPM8 overexpressing has inhibitory effects on the proliferation and metastasis of HCC cells in vitro and in vivo

**FIGURE 3:**
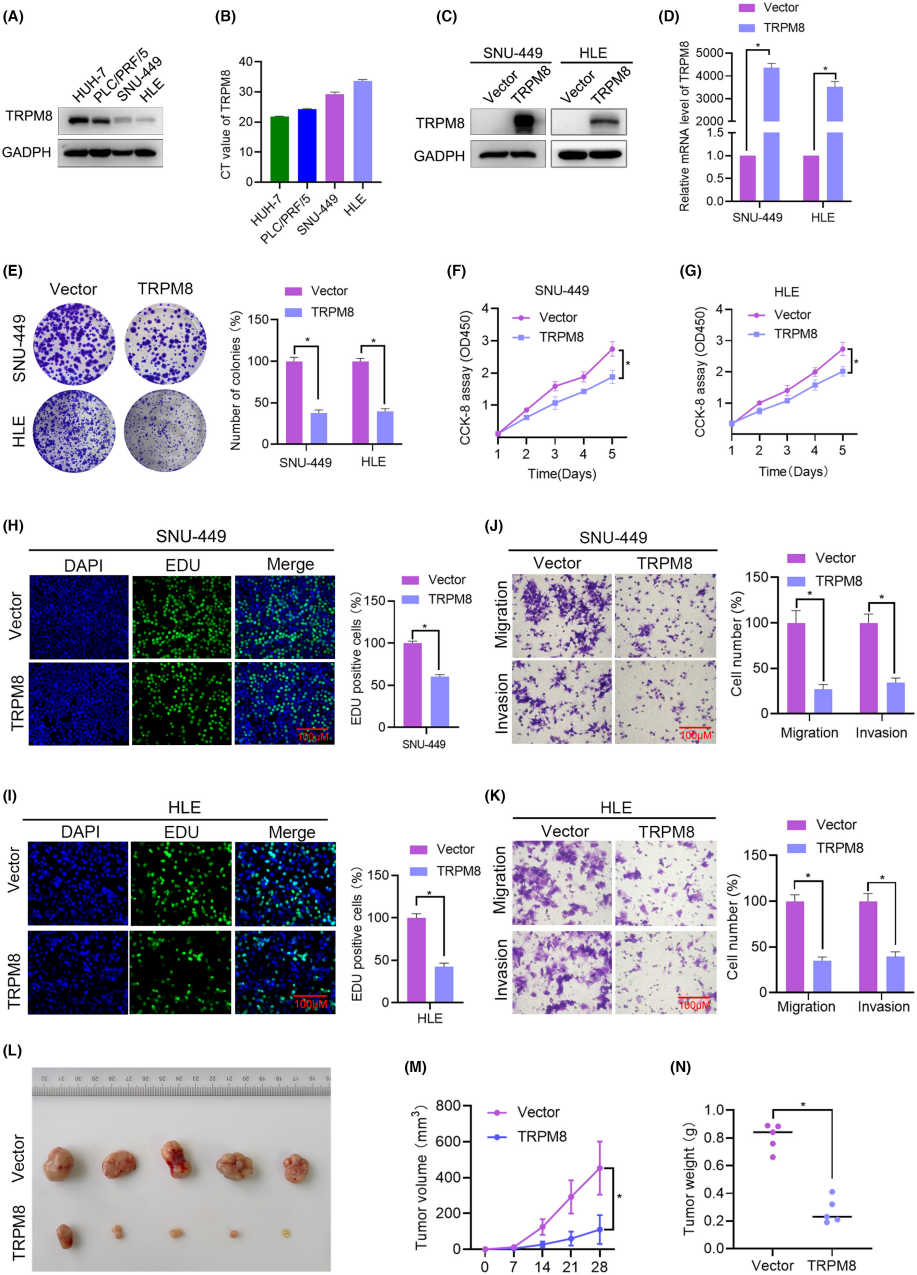
Effect of TRPM8 in inhibiting HCC cell proliferation in vitro and in vivo. (A, B) The protein and mRNA level of TRPM8 in HCC cells as determined using western blot and qRT‐PCR analyses, respectively. (C, D) TRPM8 overexpression was confirmed by western blot and qRT‐PCR after transfection with TRPM8‐overexpressing plasmids. (E–G) The effect of TRPM8 overexpression on HCC cell proliferation was determined by colony formation assays (E) and CCK‐8 (F, G). (H, I) SNU‐449 and HLE cells were seeded onto coverslips and DNA synthesis was assessed via EdU immunofluorescence staining. The graph on the left depicts the percentage of EdU‐positive nuclei. (J, K) The effects of TRPM8 overexpression on the cell migration and invasion of SNU‐449 and HLE cells were detected using Transwell assays. (L–N) Effect of TRPM8 gain‐of‐function in SNU‐449 cells on subcutaneous tumour growth (L). Tumour volumes (M). The mice were sacrificed 28 d after cell injection and the tumours were removed and weighed (N). Scale bars: 100 μm. *p < 0.05.

Endogenous TRPM8 mRNA and protein levels in different HCC cell lines were detected using qRT‐PCR and western blotting, respectively, to determine the effect of TRPM8 on the proliferation of HCC cells in vitro. The results showed that TRPM8 expression was higher in HUH‐7 and PLC/PRF/5 cells than in SNU‐449 and HLE cells (Figure 3A,B). Therefore, considering the relatively low levels of TRPM8 in SNU‐449 and HLE cell lines, we selected these two cell lines for transfection with the TRPM8 pcDNA3.1‐EGFP‐C2 plasmid. The expression of TRPM8 in the treated HCC cell lines was confirmed through western blotting (Figure 3C) and qRT‐PCR (Figure 3D). Crucially, TRPM8 overexpressing SNU‐449 and HLE cells proliferated significantly slower than control cells, as evidenced by colony formation (Figure 3E) and CCK‐8 assays (Figure 3F,G). EdU staining of TRPM8 overexpressing cells revealed that the ratio of EdU‐positive nuclei in these cells was lower than that in control cells (Figure 3H,I). TRPM8 overexpression attenuated the migratory and invasive capacities of SNU‐449 (Figure 3J) and HLE cells (Figure 3K). These results suggest that TRPM8 overexpression plays an anti‐tumour role in HCC in vitro.

To confirm the suppression of HCC by TRPM8, stable TRPM8‐overexpressing cells, and control cells were subcutaneously implanted near the thigh region of nude mice. In accordance with our in vitro results, both tumour volume and weight were effectively suppressed in mice injected with TRPM8 overexpressing cells (Figure 3L–N). These observations support that TRPM8 may function as a tumour suppressor in HCC.

### 3.4 TRPM8 silencing contributes to the proliferation and metastasis of HCC cells

**FIGURE 4:**
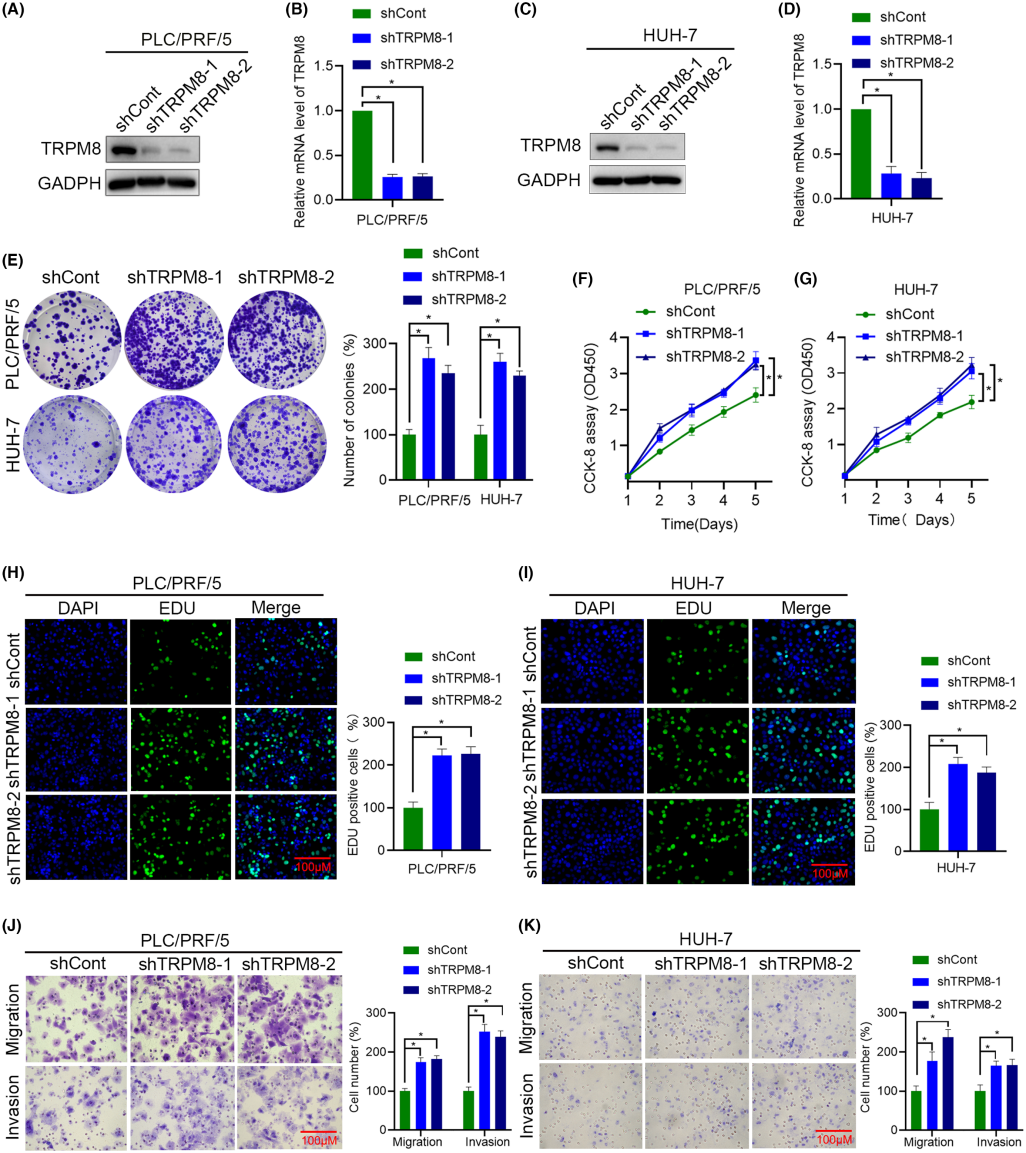
TRPM8 level determination and TRPM8‐silencing cell function experiments. (A–D) TRPM8 inhibition in PLC/PRF/5 and HUH‐7 cells transfected with shTRPM8 lentivirus was verified by Western blot and qRT‐PCR. (E–I) Effect of TRPM8 loss‐of‐function on PLC/RPF/5 and HUH‐7 cell proliferation was determined by colony formation assay (E), CCK‐8 (F‐G) and EdU assay (H, I). (J, K) The effects of TRPM8 inhibition on the cell migration and invasion of PLC/PRF/5 and HUH‐7 cells were detected using Transwell assays. Scale bars: 100 μm. *p < 0.05.

Based on the results for the above four cell lines (Figure 3A,B), we selected PLC/PRF/5 and HUH‐7 cell lines (TRPM8 high background) for transfection with shTRPM8 lentiviruses. The expression of TRPM8 in these treated HCC cell lines was examined by western blotting and qRT‐PCR (Figure 4A–D). These results suggest that the expression level of TRPM8 decreased, and the knockdown effect was significant. Consistent with the results observed in TRPM8 overexpressing SNU‐449 and HLE cells, TRPM8 silencing HUH‐7 and PLC/PRF/5 cells showed enhanced colony formation, cell viability and DNA synthesis (Figure 4E–I). In addition, the migration and invasion abilities of TRPM8 knockdown cells was greatly enhanced compared to control cells (Figure 4K). These results suggest that elevated TRPM8 levels exert an anti‐tumour effect by inhibiting the proliferation, migration and invasion capacities of HCC cells.

### 3.5 TRPM8 regulated the RTP3/STAT3 pathway in HCC

**FIGURE 5:**
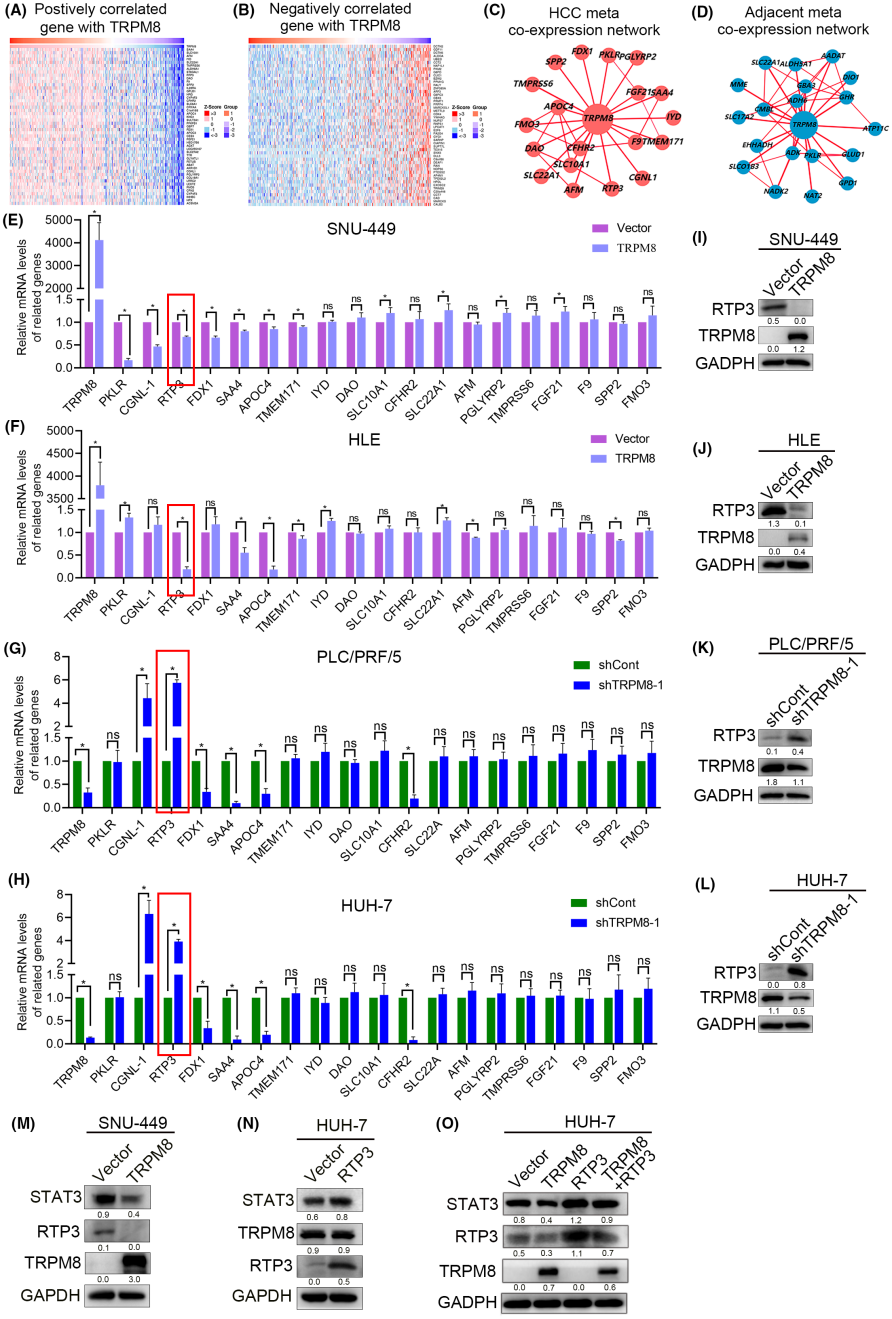
TRPM8 regulated the RTP3/STAT3 pathway in HCC. (A‐B) Heat map showed 50 genes positively correlated gene (A) and negatively correlated gene with TRPM8 (B). (C) The co‐expression network of TRPM8 in hepatocellular carcinoma. (D) The co‐expression network of TRPM8 in adjacent liver tissues. (E–H) TRPM8‐related genes were screened by qRT‐PCR and RTP3 was found. (I–L) RTP3 protein expression in TRPM8 overexpressing and silencing cells. (M) The effects of TRPM8 overexpressing HCC cells on the levels of RTP3 and STAT3 were detected by Western blot. (N) The effects of RTP3 overexpressing HCC cells on the levels of STAT3 and TRPM8 were detected by Western blot. (O)Western blot analysis of RTP3/STAT3 axis in TRPM8‐overexpressing HCC cells, and RTP3 overexpression reversed the effect ofTRPM8 on the STAT3.

To analyse the biological function of TRPM8 and identify genes positively and negatively associated with TRPM8, RNA‐seq data from the liver cancer dataset of the LinkedOmics database were used. Heat map analysis revealed 50 genes with the strongest correlations with TRPM8, both positively and negatively (Figure 5A,B). Using the HCCDB, we analysed the co‐expression network of TRPM8 and found significant differences between HCC tissues and adjacent tissues (Figure 5C,D). We then used all of the above databases to comprehensively analyse the protein–protein interactions of TRPM8. The results showed that interactions between TRPM8 and PKLR, CGNL‐1, RTP3, FDX1, SAA4, APOC4, TMEM171, IYD, DAO, SLC10A1, CFHR2, SLC22A, AFM, PGLYRP2, TMPRSS6, FGF21, F9, SPP2 and FMO3 were the strongest. To further verify these results, we performed qRT‐PCR and found that TRPM8 overexpression markedly reduced the expression of RTP3 in HCC (Figure 5E,F). In contrast, TRPM8 silencing HUH‐7 and PLC/PRF/5 cells showed the opposite effects on RTP3 expression (Figure 5G,H). This result was consistent with the western blot results (Figure 5I–L). These results indicate that RTP3 may play a role in TRPM8 mediated‐HCC progression.

Considering the tumorigenic effect of STAT3 in HCC, we hypothesised that STAT3 is involved in the TRPM8/ RTP3‐mediated signalling pathways. To verify whether TRPM8 overexpression exerts an anti‐tumour effect in HCC via inhibition of the STAT3 pathway, we treated SNU449 cells with TRPM8 overexpression and observed a repression of RTP3 and STAT3 in HCC (Figure 5M). In contrast, in HUH‐7 cells, forced expression of RTP3 exhibited the opposite effect on the STAT3 pathway but did not affect TRPM8 expression (Figure 5N). In addition, to verify whether TRPM8 overexpression in HCC exerted anti‐tumour effects by blocking the RTP3/STAT3 pathway, we performed rescue experiments to determine if reactivation of RTP3 reversed the anti‐tumour effects induced by TRPM8 overexpression (Figure 5O). We found that the reactivation of RTP3 reversed the inhibitory effect of TRPM8 on STAT3 in TRPM8‐overexpressing HCC cells. These results suggest that TRPM8 exerts anti‐tumour effects by blocking the RTP3/STAT3 signalling pathway.

### 3.6 AD80 promoted the expression of TRPM8 and inhibited the proliferation, migration and invasion of HCC cells in vitro

**FIGURE 6:**
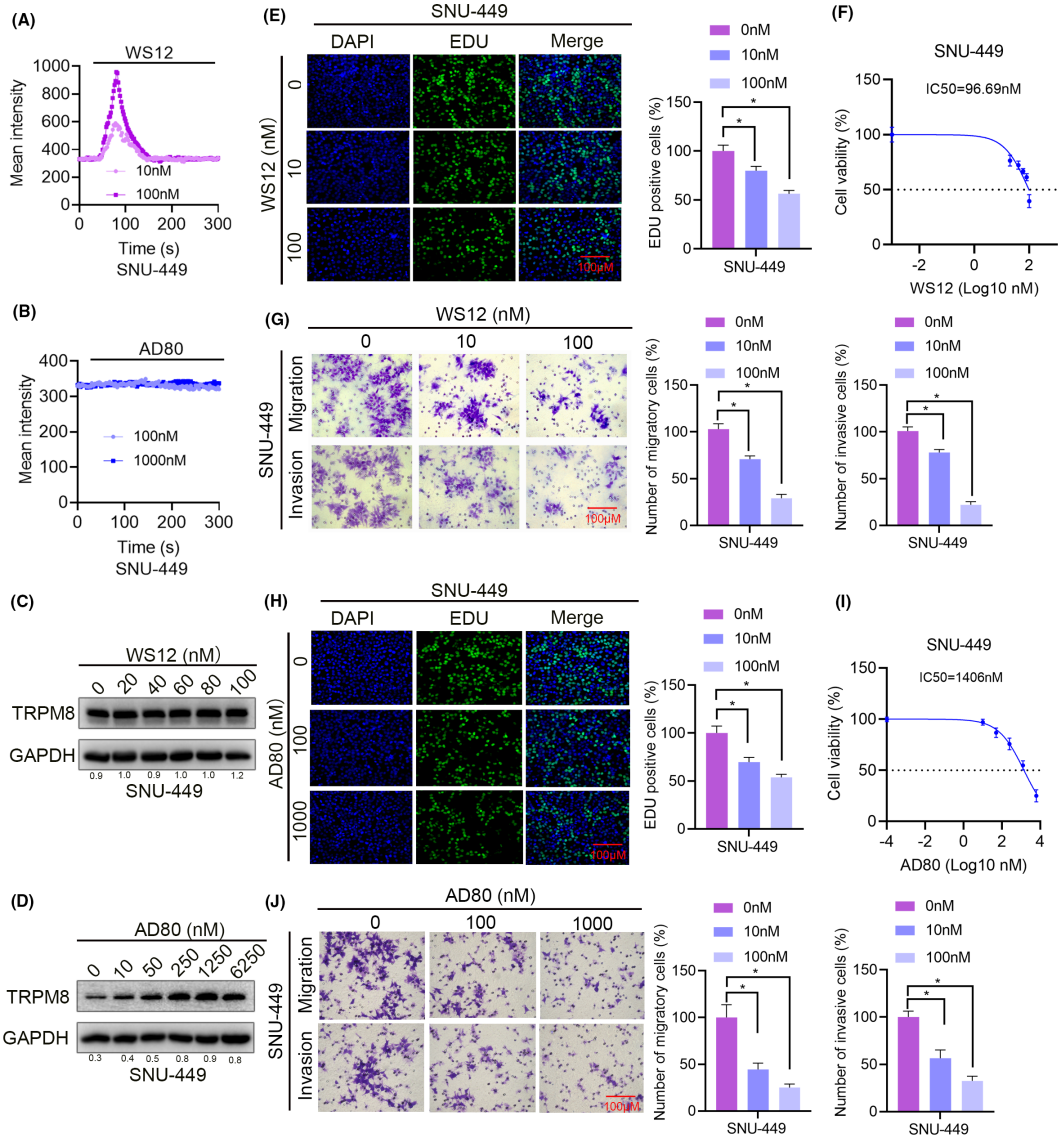
AD80 promoted the expression of TRPM8 and inhibited the proliferation, migration and invasion of HCC cells in vitro. (A, B) Calcium imaging was used to detected the changes of the fluorescence intensity of WS12 (A) and AD80 (B) groups on time in SNU‐449 cells. (C, D) Effects of WS12 (C) and AD80 (D) on the expression of TRPM8 in SNU‐449 cells were detected by Western blot. (E‐G) EdU (E) and CCK‐8 assays (F) were used to detect the effect of WS12 on the proliferation of SNU‐449. (G) The effects of WS12 on the cell migration and invasion of SSNU‐449 cells were detected using Transwell assays. (H–J) EdU (H) and CCK‐8 assays (I) were used to detect the effect of WS12 on the proliferation of SSNU‐449. (J) The effects of AD80 on the cell migration and invasion of SNU‐449 cells were detected using Transwell assays. Scale bars: 100 μm. *p < 0.05.

Based on the tumour‐suppressive role of TRPM8 in HCC, we want to explore whether AD80 could upregulate TRPM8 protein. Previous reports demonstrated that WS12 are the reported activators of TRPM8,^29^ but AD80 has never been reported before. To elucidate whether AD80 could serve as a new agonist of TRPM8, WS12‐treated cells were used as a positive control. Calcium imaging was used to determine the changes in intracellular calcium before and after AD80 treatment. The results showed that WS12, but not AD80, upregulated intracellular calcium, suggesting that AD80 may act by affecting some components of the TRPM8 synthesis process rather than by activating TRPM8 channel activity (Figure 6A,B, Figure S4A,B). We next sought to determine whether AD80 upregulates TRPM8 protein expression. The results showed that WS12 have no effects on the protein expression of TRPM8, though WS12 are agonists of TRPM8 channel proteins (Figure 6C, Figure S4C). Interestingly, AD80 activated TRPM8 expression in a dose‐dependent manner (Figure 6D, Figure S4D). In addition, to verify whether TRPM8 agonist WS12 affect the function of HCC cells, EdU, CCK‐8 and Transwell assays were performed on cells treated with WS12. The results showed that WS12 had inhibitory effects on proliferation, migration and invasion (Figure 6E–G, Figure S4E–G). Interestingly, AD80 also significantly repressed the proliferation, migration and invasion of HCC cells in a dose‐dependent manner (Figure 6H–J, Figure S4H–J). The above results suggest that AD80 may inhibit HCC cell proliferation and migration by upregulating TRPM8 protein expresson.

## 4 DISCUSSION

**FIGURE 7:**
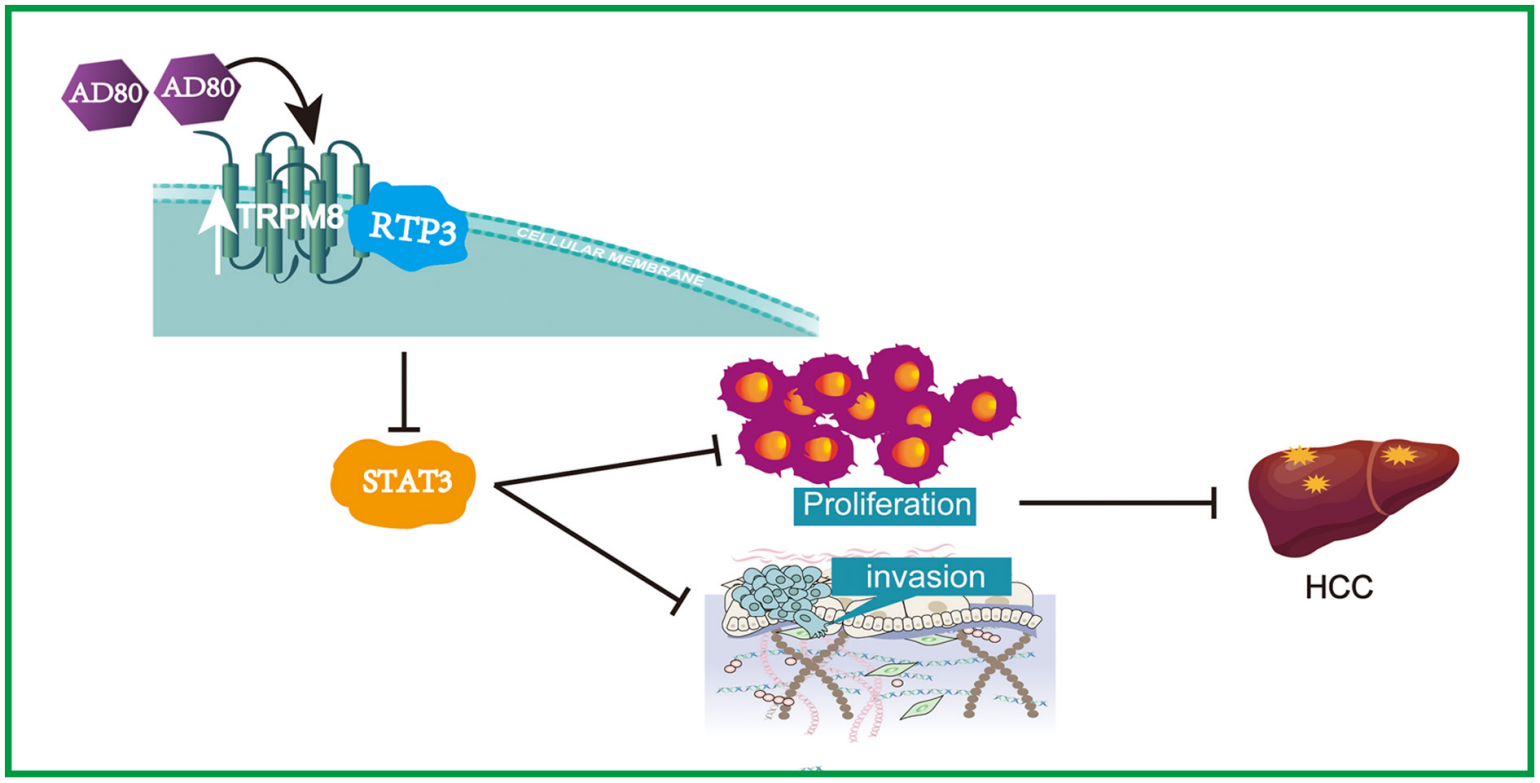
Schematic depicting the possible mechanism of the TRPM8‐mediated RTP3/STAT3 pathway in HCC.

This study shows that TRPM8 expression is low in HCC. Low TRPM8 expression predicts poor OS. AD80, a multikinase inhibitor, significantly promotes the expression of TRPM8. High TRPM8 expression inhibits the proliferation and invasion of HCC cells by influencing the RTP3/STAT3 pathway (Figure 7). Based on these compelling results, our study suggests a critical role for TRPM8 in HCC.

However, Fu et al.^30^ found that TRPM8 expression was increased in HCC and TRPM8 silencing could inhibit HCC development, which is inconsistent with our results. Indeed, this is not the first time that TRPM8 has been found to be differentially expressed in the same type of cancer. Yapa et al. extensively discussed the issue of differential expression of TRPM8 in breast cancer.^31^ They suggested that many factors, including quantitative qRT‐PCR primers, antibodies, specific cell lines and the source and batch of foetal bovine serum used for cell culture may alter TRPM8 gene expression. For this issue, we will talk about it from two main aspects. First, we determined the expression of TRPM8 in TCGA database, and the results showed that TRPM8 expression was much higher in normal tissue than in HCC. Similarly, in GSE25097, GSE54236 and GSE76427 datasets, TRPM8 expression was also found to be higher in normal tissues than in HCC tissues. Consistent with the above data, we found that in the tissue samples we collected from HCC patients, TRPM8 expression was higher in normal tissues than in HCC tissues. Furthermore, low expression of TRPM8 was found to be associated with clinicopathological features and poor prognosis of HCC patients. Fu and colleagues used only tissue samples from HCC patients in their cohort. Therefore, the differences between the two groups may be due to individual differences in HCC patients as well as significant tissue heterogeneity. In addition, the number of patient cohorts in the other two studies may be insufficient and more cases need to be collected. Second, the cell lines used in these two reports were different. In our study, we found that TRPM8 expression was significantly reduced in SNU‐449 and HLE, and relevant functional experiments were carried out in these two cell models. Hence, the differential expression of TRPM8 may be due to the use of different cell types or different sources.

Dysregulation of TRPPM8 has been observed in various cancers and may promote tumour proliferation and metastasis. More studies have shown that TRPM8 plays an important role in cancer progression by affecting the epithelial‐mesenchymal transition (EMT) process. EMT refers to the loss of typical epithelial cell characteristics and the acquisition of a mesenchymal phenotype in epithelial cells, which is considered to be a pathological process of tumour progression. It is regulated by a variety of signal transduction pathways, including PI3K/AKT, NF‐κB, JAK/STAT3 and AKT/GSK‐3β/Snail,^32^ and is a major mechanism of invasion and metastasis.^33^ In recent years, transient receptor potential (TRP) channels have attracted much attention in cancer targeted therapy.^34^ In cancer cells, the channel is often altered, and once its normal function is disrupted, it can lead to malignant tumour progression and growth by affecting a variety of signalling pathways. TRPM8, a member of the TRP family, has also been found to be associated with EMT and plays an important role in cancer progression.^35^ In addition, knockdown of TRPM8 has been reported to reduce the migration and invasion ability of A498 renal cancer cells, and it is speculated that TRPM8 may regulate EMT by regulating the expression of snail and WNT‐5a, the EMT upstream signalling molecules, thereby affecting cell migration and invasion.^35^ Furthermore, previous studies have shown that increasing TRPM8 can enhance the activity of MMP‐9 and promote the activation of PI3K/AKT signalling pathway, thereby playing a positive role in the process of EMT.^36^ At the same time, it was found that TRPM8 was strongly expressed in the early stage of prostate cancer and disappeared in the late stage and more aggressive state of prostate cancer.^37^ Due to the change in its expression, TRPM8 can be considered a diagnostic or prognostic marker. Similarly, TRPM8 is abnormally overexpressed in pancreatic cancer cell lines and tissues and various histopathological types of pancreatic tumours, with TRPM8 channels playing a role in tumour growth, invasion and metastasis.^38^ Furthermore, menthol, an agonist of TRPM8, induces human bladder cancer T‐cell death through TRPM8.^39^ Wang et al. studied the effects of TRPM8 on osteosarcoma. The results showed that TRPM8 was highly expressed in human osteosarcoma cells and osteochondroma specimens. Deletion of TRPM8 inhibited cell proliferation, migration and invasion. In addition, although TRPM8 knockdown does not trigger apoptosis, it promotes EPI‐induced apoptosis.^40^ These studies show that TRPM8 is a key regulator of osteosarcoma and may serve as a clinical biomarker and therapeutic target for osteosarcoma. At present, the role of TRPM8 in HCC is unclear; however, because of its important role in various cancers, we speculate that TRPM8 may affect HCC.

Based on our analysis, we found that TRPM8 is involved in various molecular metabolic processes in HCC cells, particularly those related to RTP3. Interestingly, another TRPM family member, TRPC1, is significantly expressed in breast cancer cells and promotes tumour invasion and resistance to chemotherapeutic agents by activating the hypoxia‐inducing factor‐1α (HIF1α) pathways mediated by EGFR, STAT3 and autophagy marker LC3B.^41^ The TMEMs are a class of proteins that can span biological membranes, including plasma and organelle membranes. Many TMEMs act as channels that permit the transport of specific substances through biofilms.^42^ Furthermore, TMEMs are present in many cell types and perform important physiological functions such as epidermal keratosis (TMEM45A)^43^ autophagy, and liver development and differentiation (TMEM97).^44^ In addition, differential regulation of TMEMs expression has been observed in many cancers such as oesophageal squamous cell cancer (TMEM16),^45^ lymphoma (TMEM176),^46^ colorectal carcinomas (TMEM25),^47^ gastric cancer (TM4SF1),^48^ lung carcinomas (TMEM48)^49^ and hepatocarcinoma (RTP3, TMEM7).^50^ Some of these biomarkers can be used as predictive biomarkers for cancer. However, the role and mechanism of RTP3 in liver cancer remain unclear, prompting further exploration into whether RTP3 could be a new molecular target for the treatment and prognosis of liver cancer.

Several TMEMs are upregulated in cancer as oncogenes and are associated with tumour progression, migration and invasion. It has previously been reported that RTP3 is similar to the zf‐3CxxC family of transcriptional regulators and is involved in the occurrence and development of renal cell carcinoma (RCC).^51^ Furthermore, Shen et al. found that TMEM45B was upregulated in gastric cancer cell lines. Its knockdown inhibits the proliferation, migration and invasion of gastric cancer cells in vitro and tumour growth in nude mice. These effects are related to TMEM45B silencing and decreased p‐STAT3 and p‐JAK2 levels.^52^ Therefore, we speculated that TRPM8 affects HCC through the RTP3/STAT3 pathway; however, the specific mechanism requires further study.

However, given the conclusion that TRPM8 negatively regulates STAT3 and that RET and SRC can activate STAT3 by inducing the phosphorylation of tyrosine residues, we turned our attention to kinase inhibitors, of which AD80 attracted our attention as a multi‐kinase inhibitor of RET, RAF, SRC and S6K.^53^, ^54^, ^55^ In our study, we found that AD80 was involved in the anti‐tumour process by upregulating the expression of TRPM8 protein. AD80 could significantly upregulate the expression of TRPM8 and inhibit the proliferation and metastasis of HCC cells.

Notely, although this study used in vitro and in vivo models to investigate the role of TRPM8, they may not fully recapitalize the complexity of human HCC due to the inherent limitations of the models and the complexity of tumour tissue. In addition, Fu et al. found that the expression of TRPM8 was increased in HCC, and inhibition of TRPM8 could repress the development of HCC.^30^ Therefore, whether TRPM8 can be served as a potential biomarker for prognosis and diagnosis of HCC remains to be determined. It must be noted that more comprehensive validation and replication studies are needed to confirm its utility as a biomarker. There may also be potential confounding variables that could affect the findings, such as other comorbidities in HCC patients.

## 5 CONCLUSIONS

TRPM8 mRNA and protein levels in HCC tissues were lower than those in normal liver and adjacent tissues, and the decrease in TRPM8 mRNA levels was related to the histological grade and short OS of patients with HCC. Our results reveal that the TRPM8‐RTP3‐STAT3 axis plays a crucial role in maintaining the malignant progression of HCC. Moreover, our work demonstrated that AD80 could upregulate the expression of TRPM8 and participate in the anti‐tumour process.

## AUTHOR CONTRIBUTIONS

**Lichan Chen:** Data curation (equal); investigation (equal); writing – original draft (equal). **Nansong Xu:** Conceptualization (equal); validation (equal); writing – review and editing (equal). **DongMei Gou:** Investigation (equal); validation (equal); writing – review and editing (equal). **Jianning Song:** Data curation (equal); resources (equal); writing – original draft (equal). **Mingqin Zhou:** Investigation (equal); validation (equal). **Yajun Zhang:** Methodology (equal); validation (equal); writing – review and editing (equal). **Haohua Zhang:** Investigation (equal); validation (equal). **Liwen Zhu:** Formal analysis (equal); validation (equal). **Weihong Huang:** Formal analysis (equal); visualization (equal). **Yue Zhu:** Methodology (equal); software (equal). **Cheng Gao:** Formal analysis (equal); funding acquisition (equal); supervision (equal). **Dayong Gu:** Methodology (equal); supervision (equal); writing – review and editing (equal). **Yong Xu:** Funding acquisition (equal); supervision (equal); writing – review and editing (equal). **Hongzhong Zhou:** Funding acquisition (equal); supervision (equal); writing – review and editing (equal).

## FUNDING INFORMATION

This work was supported by National Key R&D Programmes (Grant No. 2022YFC2302700); National Natural Science Foundation of China (Grant No. 82203870); Shenzhen Medical Research Funds (Grant No. A2303053); Shenzhen Science and Technology Program (Grant No. ZDSYS20210623092001003; KCXFZ20230731094904008; JCYJ20210324103407018; GJHZ20200731095604013; JSGG20220301090003004; No. 201906133000069; No. SGLH20180625171602058; No. RCBS20200714114856016; No. JCYJ20210324103204011; No. GJHZ20200731095604013); Basic Research General Program of Shenzhen Science and Technology Innovation Commission (Grant No. JCYJ20190809160001751); the Guangdong Science and Technology Foundation (No. 2020A1515110871); Guangdong Basic and Applied Basic Research Foundation (Grant No. 2021A1515110931; No. 2021A1515220084; No. 2020B1111160001); Shenzhen Third People's Hospital (Grant No. 23260G1001).

## CONFLICT OF INTEREST STATEMENT

The authors declare that the research was conducted in the absence of any commercial or financial relationships that could be construed as a potential conflict of interest.

## INSTITUTIONAL REVIEW BOARD STATEMENT

The study was conducted in accordance with the Declaration of Helsinki, and approved by the Ethics Committee of the Shenzhen Second People's Hospital (protocol code: 2023–262‐01, data of approval: 14 November 2023). All animal experiments were approved by the Shenzhen Second People's Hospital (protocol code: 202300208, data of approval: 15 November 2023), and all procedures were performed in accordance with the institutional guidelines.

## INFORMED CONSENT

Informed consent was obtained from all subjects or their relatives.

## Supporting information


Figure S1.

Figure S2.

Figure S3.

Figure S4.

Table S1.

## Data Availability

Data sets for the study period and/or the analysis period may be obtained from the corresponding author upon reasonable request.
