# Supplementary figures and images for: TRPM8 overexpression suppresses hepatocellular carcinoma progression and improves survival by modulating the RTP3/STAT3 pathway
Source: Cancer Med. 2024 Oct 9;13(19):e70109. doi: 10.1002/cam4.70109 (PMC11464657; doi:10.1002/cam4.70109)

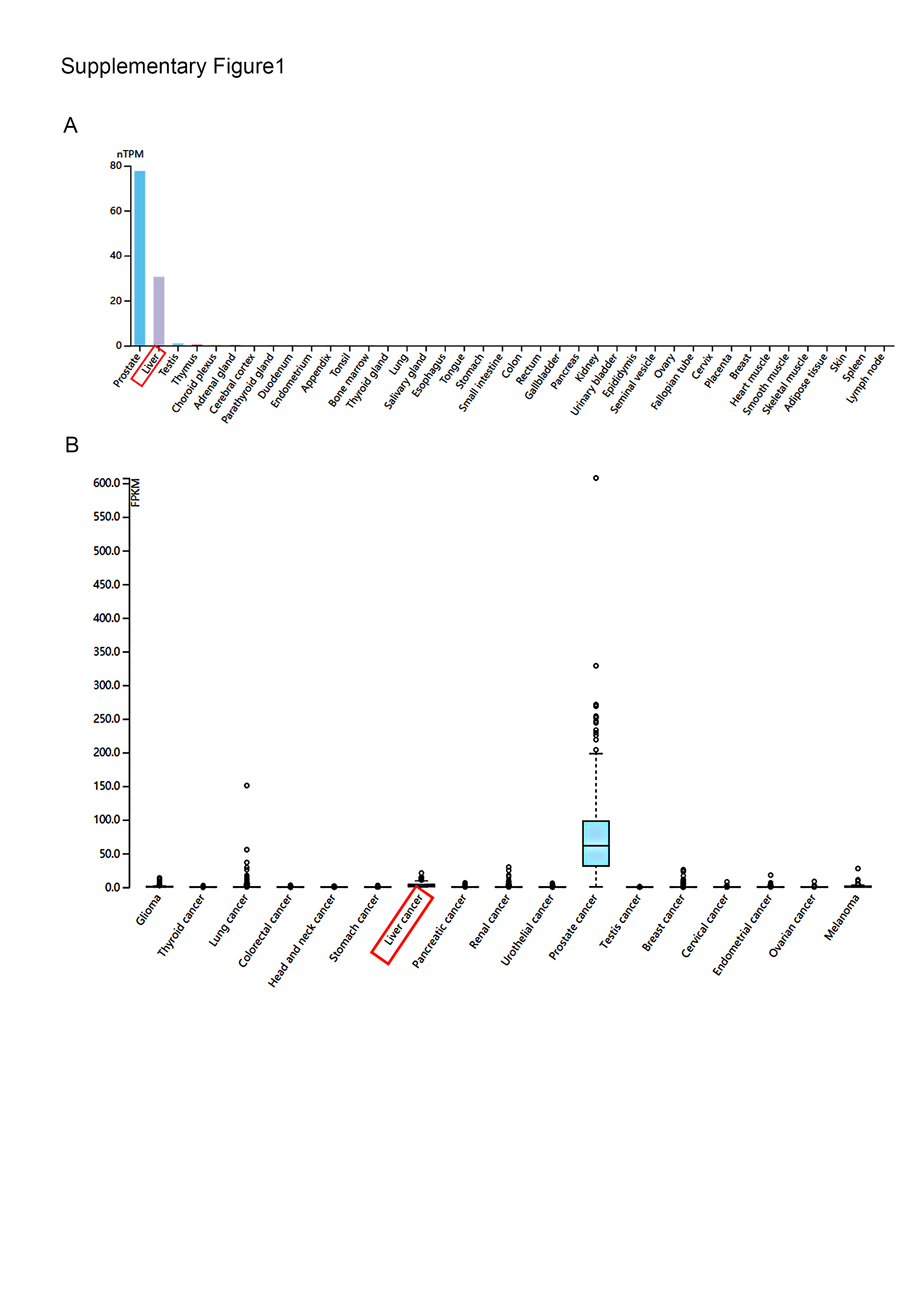

Supplement: Supplementary file 1 — Figure S1. [file CAM4-13-e70109-s005.tif]

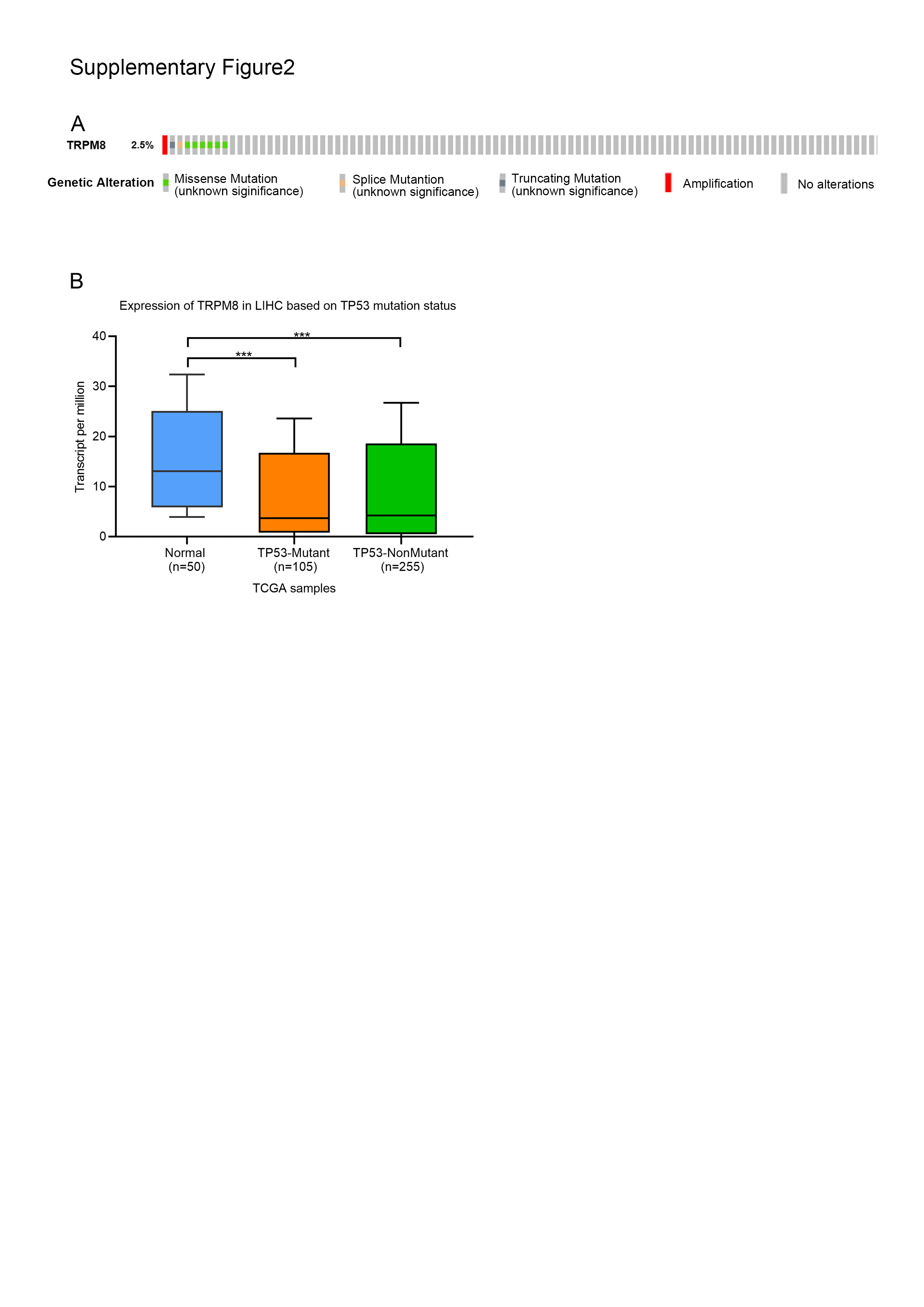

Supplement: Supplementary file 2 — Figure S2. [file CAM4-13-e70109-s004.tif]

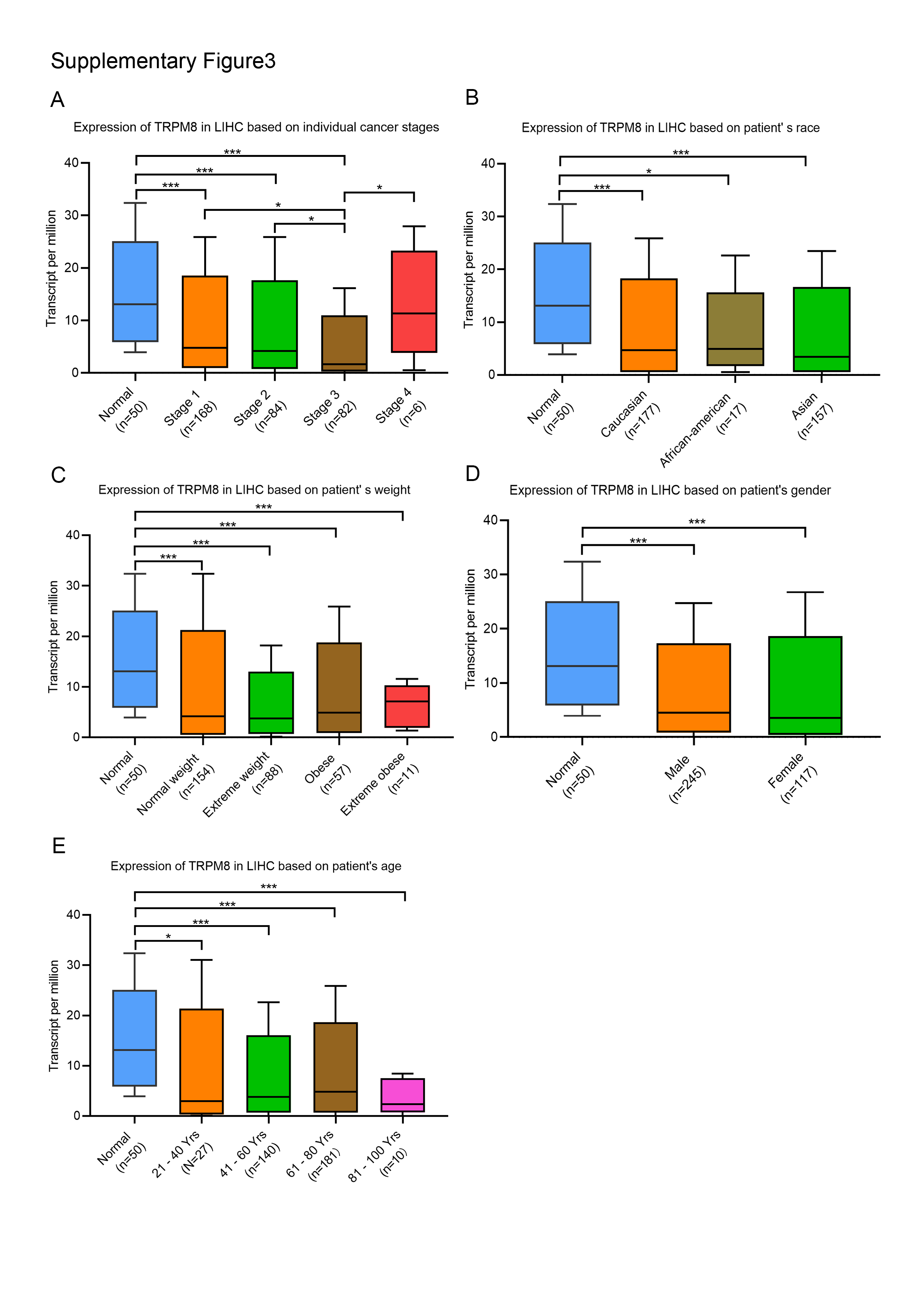

Supplement: Supplementary file 3 — Figure S3. [file CAM4-13-e70109-s002.tif]

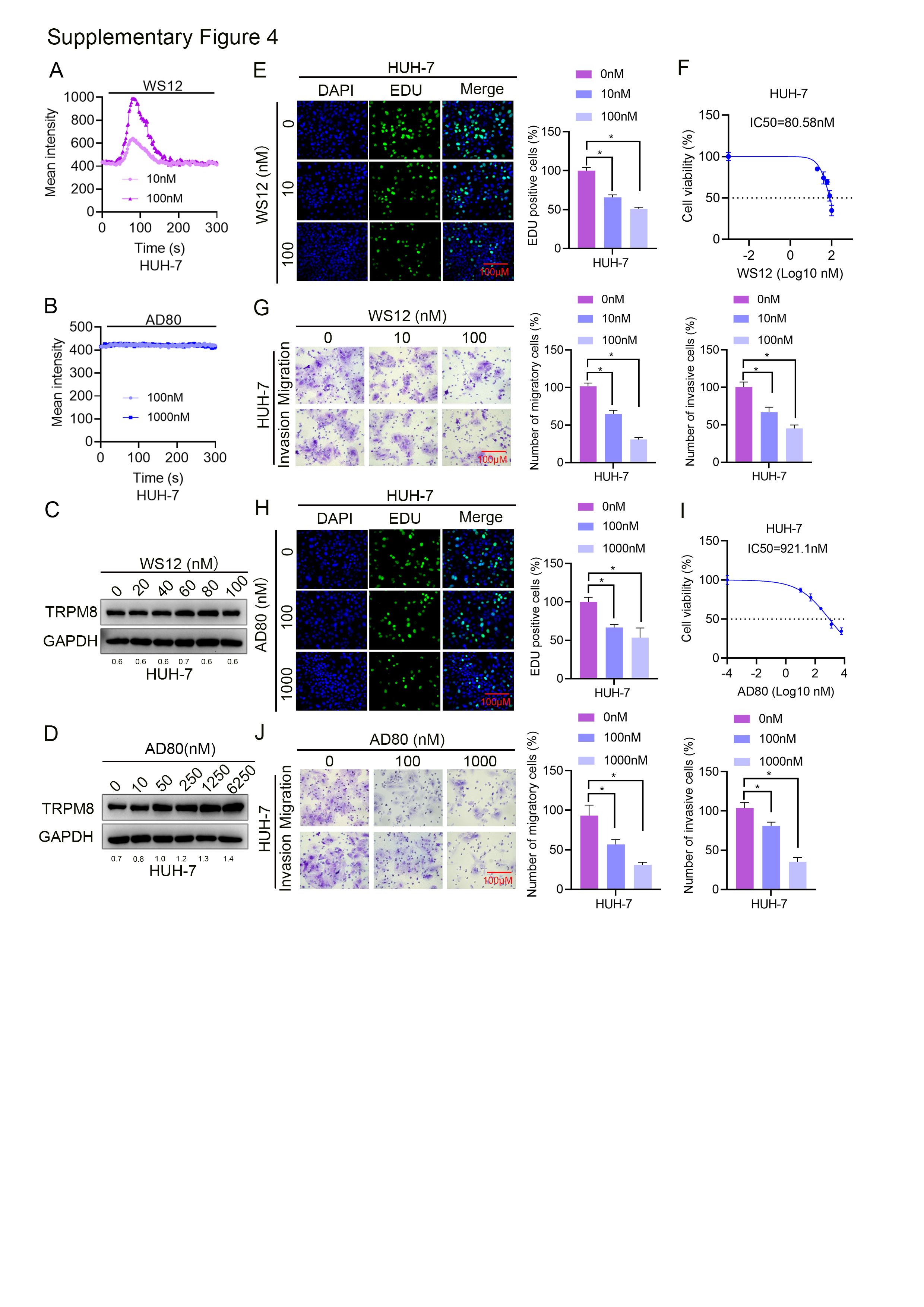

Supplement: Supplementary file 4 — Figure S4. [file CAM4-13-e70109-s003.tif]
